# Excursion to New Orleans in May Next

**Published:** 1869-02

**Authors:** James F. H. Hibberd

**Affiliations:** Richmond, Ind.


### Excursion to New Orleans in May next.

RICHMOND, IND., Feb'y 19, 1869.

Editor Buffalo Medical and Surgical Journal:

At Washington, in May last, when the American Medical Association decided to hold its next session at New Orleans, I promised several gentlemen to see if a special arrangement could not be made with a large, first class steamer, to take all those who might wish to go down the Mississippi from Cairo to New Orleans, as a kind of excursion party. My correspondence with the steamboat men warrants me in saying that such an arrangement can be made, and I shall be able to furnish particulars for publication by the first of April. Meanwhile I thought perhaps your readers might be interested to know that such a negotiation was being prosecuted.

The time from Cairo to New Orleans will be about four days, and the fare from Cairo to New Orleans and return will not exceed thirty-five dollars. This includes meals and state-rooms. Perhaps better than this can be done, but as soon as I have the affair definitely understood I will advise you.

Prof. Gross and others thought that all who were going via the river, by getting on one good, well ordered boat, might make the voyage both pleasant and profitable.

Very truly, etc., JAMES F. H. HIBBERD.

